# Amos-type bounds for modified Bessel function ratios^☆^

**DOI:** 10.1016/j.jmaa.2013.05.070

**Published:** 2013-12-01

**Authors:** Kurt Hornik, Bettina Grün

**Affiliations:** a Institute for Statistics and Mathematics, WU Wirtschaftsuniversität Wien, Augasse 2–6, 1090 Vienna, Austria; b Department of Applied Statistics, Johannes Kepler University Linz, Altenbergerstraße 69, 4040 Linz, Austria

**Keywords:** Bounds, Modified Bessel function ratio, Modified Bessel functions of the first kind, Inequalities

## Abstract

We systematically investigate lower and upper bounds for the modified Bessel function ratio $R_{\nu }=I_{\nu +1}/I_{\nu }$ by functions of the form $G_{\alpha ,\beta }\left(t\right)=t/\left(\alpha +\sqrt{t^{2}+\beta^{2}}\right)$ in case $R_{\nu }$ is positive for all t>0, or equivalently, where ν≥−1 or ν is a negative integer. For ν≥−1, we give an explicit description of the set of lower bounds and show that it has a greatest element. We also characterize the set of upper bounds and its minimal elements. If ν≥−1/2, the minimal elements are tangent to $R_{\nu }$ in exactly one point 0≤t≤∞, and have $R_{\nu }$ as their lower envelope. We also provide a new family of explicitly computable upper bounds. Finally, if ν is a negative integer, we explicitly describe the sets of lower and upper bounds, and give their greatest and least elements, respectively.

## 1 Introduction

Let $I_{\nu }$ be the modified Bessel function of order ν, and $R_{\nu }$ the (modified) Bessel function ratio $R_{\nu }\left(t\right)=I_{\nu +1}\left(t\right)/I_{\nu }\left(t\right)$. These ratios are of great importance in a variety of application areas, including statistics  [e.g., 7] and numerical analysis  [e.g., 1], either directly or through the fact that by the well-known recurrence relations for modified Bessel functions,

$$\log{\left(I_{\nu }\right)}^{\prime }\left(t\right)=\frac{I_{\nu }^{\prime }\left(t\right)}{I_{\nu }\left(t\right)}=\frac{I_{\nu +1}\left(t\right)+\left(\nu /t\right)I_{\nu }\left(t\right)}{I_{\nu }\left(t\right)}=R_{\nu }\left(t\right)+\frac{\nu }{t}$$

from which by integration and taking limits,

$$\log\left(I_{\nu }\right)\left(t\right)=\int_{0}^{t}R_{\nu }\left(s\right)\;ds+\nu \log\left(t/2\right)-\log\left(\Gamma \left(\nu +1\right)\right)\text{.}$$

For functions f and g defined on the positive reals, write f≤g iff f(t)≤g(t) for all t>0, with f<g defined analogously. If neither f≤g nor g≤f, we say that f and g are incomparable. Let G be a family of functions on the positive reals and f∈G. We say that f is the least element (minimum) of G iff f≤g for all g∈G, and that f is a minimal element of G iff there is no g∈G for which f>g, with the greatest element (maximum) and maximal elements of G defined analogously.

Let

$$G_{\alpha ,\beta }\left(t\right)=\frac{t}{\alpha +\sqrt{t^{2}+\beta^{2}}}\text{,}$$

where in what follows we always (without loss of generality) take β≥0. For ν≥0, Eqs. (9), (11) and (16) in Amos [1] show that

$$\max\left(G_{\nu +1,\nu +1},G_{\nu +1/2,\nu +3/2}\right)\leq R_{\nu }\leq \min\left(G_{\nu ,\nu },G_{\nu ,\nu +2},G_{\nu +1/2,\nu +1/2}\right)\text{.}$$

Such “Amos-type” bounds were re-established and extended in several publications (see Section  3 for details). These bounds are very attractive because they allow both for explicit inversion and integration. Thus, Amos-type bounds yield bounds (and approximations) also for $R_{\nu }^{-1}$ and the antiderivate of $R_{\nu }$ (equivalently, $I_{\nu }$ and its logarithm).

Let

$$L_{\nu }=\left\{\left(\alpha ,\beta \right):G_{\alpha ,\beta }\leq R_{\nu }\right\}\text{,}\;U_{\nu }=\left\{\left(\alpha ,\beta \right):G_{\alpha ,\beta }\geq R_{\nu }\right\}$$

be the set of all (α,β) for which $G_{\alpha ,\beta }$ is a lower/upper Amos-type bound for $R_{\nu }$, and write

$$G_{L_{\nu }}=\left\{G_{\alpha ,\beta }:\left(\alpha ,\beta \right)\in L_{\nu }\right\}\text{,}\;G_{U_{\nu }}=\left\{G_{\alpha ,\beta }:\left(\alpha ,\beta \right)\in U_{\nu }\right\}\text{,}$$

for the corresponding families of lower/upper Amos-type bounds for $R_{\nu }$.

In this paper, we investigate the structure of $G_{L_{\nu }}$ and $G_{U_{\nu }}$ under the condition that $R_{\nu }>0$, or equivalently, ν≥−1 or ν a negative integer.

## 2 Preliminaries

Let

$$v_{\nu }\left(t\right)=tI_{\nu }\left(t\right)/I_{\nu +1}\left(t\right)=t/R_{\nu }\left(t\right)$$

and

$$h_{\alpha ,\beta }\left(t\right)=\alpha +\sqrt{t^{2}+\beta^{2}}$$

so that $G_{\alpha ,\beta }\left(t\right)=t/h_{\alpha ,\beta }\left(t\right)$.

Using, e.g., Watson  [10, Formula 3.7.2],

$$R_{\nu }\left(t\right)=\frac{t}{2}\frac{\overset{\infty }{\underset{n=0}{\sum }}t^{2n}/\left(4^{n}n!\Gamma \left(n+\nu +2\right)\right)}{\overset{\infty }{\underset{n=0}{\sum }}t^{2n}/\left(4^{n}n!\Gamma \left(n+\nu +1\right)\right)}\text{.}$$

If ν≥−1, all coefficients in the numerator and denominator series are non-negative and eventually positive, and hence $R_{\nu }>0$. If ν is a negative integer, the same is true; otherwise, $\lim_{t\to 0}v_{\nu }\left(t\right)=2\Gamma \left(\nu +2\right)/\Gamma \left(\nu +1\right)=2\left(\nu +1\right)$ which is negative if ν<−1, and hence $R_{\nu }\left(t\right)<0$ for all sufficiently small positive t.

Using the asymptotic expansion of $I_{\nu }$ for large argument [10, e.g., Formula 7.23.2], one can show that for arbitrary  ν,

(1) $$R_{\nu }\left(t\right)=1-\frac{\nu +1/2}{t}+\frac{\nu^{2}-1/4}{2t^{2}}+O\left(1/t^{3}\right)\text{,}\;t\to \infty \text{,}$$

see also Schou  [7, Eq. (6), assuming  ν≥0].

As $h_{\alpha ,\beta }$ is increasing with $h_{\alpha ,\beta }\left(0\right)=\alpha +\beta$, we have $G_{\alpha ,\beta }>0$ iff α+β≥0. Hence, when ν≥−1 or ν is a negative integer and $\alpha +\beta \geq 0,G_{\alpha ,\beta }$ is a (strict) upper or lower bound for $R_{\nu }$ if and only if $h_{\alpha ,\beta }$ is a (strict) lower or upper bound for $v_{\nu }$, respectively.

Lemma 1 *For* ν≥−1*,*

(2) $$v_{\nu }\left(t\right)=2\left(\nu +1\right)+\frac{t^{2}}{2\left(\nu +2\right)}+O\left(t^{4}\right)\text{,}\;t\to 0\text{.}$$

Proof More generally, if ν is not a negative integer,

$$v_{\nu }\left(t\right)=t\frac{{\left(t/2\right)}^{\nu }\left(\frac{1}{\Gamma \left(\nu +1\right)}+\frac{t^{2}/4}{\Gamma \left(\nu +2\right)}+O\left(t^{4}\right)\right)}{{\left(t/2\right)}^{\nu +1}\left(\frac{1}{\Gamma \left(\nu +2\right)}+\frac{t^{2}/4}{\Gamma \left(\nu +3\right)}+O\left(t^{4}\right)\right)}=2\frac{\left(\nu +1\right)+\frac{t^{2}}{4}+O\left(t^{4}\right)}{1+\frac{t^{2}}{4\left(\nu +2\right)}+O\left(t^{4}\right)}=2\left(\nu +1\right)+\frac{t^{2}}{2\left(\nu +2\right)}+O\left(t^{4}\right)\text{,}\;t\to 0\text{.}$$

If ν=−k is a negative integer, 1/Γ(ν+n+1) vanishes for n from 0 to k−1, and hence

$$v_{-k}\left(t\right)=2\frac{\overset{\infty }{\underset{n=k}{\sum }}t^{2n}/\left(4^{n}n!\Gamma \left(n-k+1\right)\right)}{\overset{\infty }{\underset{n=k-1}{\sum }}t^{2n}/\left(4^{n}n!\Gamma \left(n-k+2\right)\right)}=2\frac{t^{2k}/\left(4^{k}k!\right)}{t^{2\left(k-1\right)}/\left(4^{k-1}\left(k-1\right)!\right)}\frac{1+\frac{t^{2}}{4\left(k+1\right)}+O\left(t^{4}\right)}{1+\frac{t^{2}}{4k}+O\left(t^{4}\right)}=\frac{t^{2}}{2k}+O\left(t^{4}\right)\text{,}\;t\to 0\text{.}$$

As for ν=−k=−1 we have 2(ν+1)=0 and ν+2=1=k, we can combine the two expansions to obtain the lemma. □

Lemma 2 *If* β>0*,*

(3) $$h_{\alpha ,\beta }\left(t\right)=\left(\alpha +\beta \right)+\frac{t^{2}}{2\beta }+O\left(t^{4}\right)\text{,}\;t\to 0\text{.}$$

*For arbitrary* α *and* β≥0*,*

(4) $$G_{\alpha ,\beta }\left(t\right)=1-\frac{\alpha }{t}+\frac{2\alpha^{2}-\beta^{2}}{2t^{2}}+O\left(t^{-3}\right)\text{,}\;t\to \infty \text{.}$$

Proof If β>0, then

$$\sqrt{t^{2}+\beta^{2}}=\beta \sqrt{1+{\left(t/\beta \right)}^{2}}=\beta \left(1+\frac{t^{2}}{2\beta^{2}}+O\left(t^{4}\right)\right)=\beta +\frac{t^{2}}{2\beta }+O\left(t^{4}\right)$$

for t→0, whence Eq. (3) by adding α.

As $t\to \infty ,\sqrt{1+\beta^{2}/t^{2}}=1+\beta^{2}/\left(2t^{2}\right)+O\left(t^{-4}\right)$ and thus

$$G_{\alpha ,\beta }\left(t\right)=\frac{1}{\alpha /t+\sqrt{1+\beta^{2}/t^{2}}}=\frac{1}{1+\alpha /t+\beta^{2}/\left(2t^{2}\right)+O\left(t^{-4}\right)}=1-\frac{\alpha }{t}+\frac{2\alpha^{2}-\beta^{2}}{2t^{2}}+O\left(t^{-3}\right)\text{,}\;t\to \infty \text{.}$$

 □

Theorem 1 *For arbitrary* $\nu ,G_{\alpha ,\beta }\leq R_{\nu }$ *or* $G_{\alpha ,\beta }\geq R_{\nu }$ *are only possible when* α≥ν+1/2 *or* α≤ν+1/2*, respectively. If* ν≥−1*, then* $G_{\alpha ,\beta }\leq R_{\nu }$ *or* $G_{\alpha ,\beta }\geq R_{\nu }$ *are only possible when* α+β≥2(ν+1) *or* 0≤α+β≤2(ν+1)*, respectively.*

Proof The first assertion is immediate by comparing the expansions of $R_{\nu }$ and $G_{\alpha ,\beta }$ for t→∞. If $\alpha +\beta <0,h_{\alpha ,\beta }$ has a unique zero t>0, and $G_{\alpha ,\beta }$ changes from −∞ to ∞ at t. If $\nu \geq -1,R_{\nu }>0$, so upper and lower $G_{\alpha ,\beta }$ bounds necessarily must have α+β≥0. The second assertion now follows by comparing the values of $v_{\nu }$ and $h_{\alpha ,\beta }$ at t=0. □

Lemma 3 *Let* $\beta_{1}<\beta_{2}$ *and* $\min\left(\alpha_{1}+\beta_{1},\alpha_{2}+\beta_{2}\right)\geq 0$ *. Then* $G_{\alpha_{1},\beta_{1}}<G_{\alpha_{2},\beta_{2}}$ *iff* $\alpha_{1}+\beta_{1}\geq \alpha_{2}+\beta_{2}$*, and* $G_{\alpha_{1},\beta_{1}}>G_{\alpha_{2},\beta_{2}}$ *iff* $\alpha_{1}\leq \alpha_{2}$ *. Otherwise, if* $\alpha_{1}>\alpha_{2}$ *and* $\alpha_{1}+\beta_{1}<\alpha_{2}+\beta_{2}$ *and*

$$t=\frac{\sqrt{\left({\left(\beta_{2}-\beta_{1}\right)}^{2}-{\left(\alpha_{1}-\alpha_{2}\right)}^{2}\right)\left({\left(\beta_{2}+\beta_{1}\right)}^{2}-{\left(\alpha_{1}-\alpha_{2}\right)}^{2}\right)}}{2\left(\alpha_{1}-\alpha_{2}\right)}\text{,}$$

$G_{\alpha_{1},\beta_{1}}\left(s\right)>G_{\alpha_{2},\beta_{2}}\left(s\right)$ *for* 0<s<t *and* $G_{\alpha_{1},\beta_{1}}\left(s\right)<G_{\alpha_{2},\beta_{2}}\left(s\right)$ *for* s>t*.*

Proof Consider $\Delta =h_{\alpha_{1},\beta_{1}}-h_{\alpha_{2},\beta_{2}}$. Then $\Delta \left(0\right)=\left(\alpha_{1}+\beta_{1}\right)-\left(\alpha_{2}+\beta_{2}\right)$ and as

$$\sqrt{t^{2}+\beta_{1}^{2}}-\sqrt{t^{2}+\beta_{2}^{2}}=\frac{\left(t^{2}+\beta_{1}^{2}\right)-\left(t^{2}+\beta_{2}^{2}\right)}{\sqrt{t^{2}+\beta_{1}^{2}}+\sqrt{t^{2}+\beta_{2}^{2}}}=\frac{\beta_{1}^{2}-\beta_{2}^{2}}{\sqrt{t^{2}+\beta_{1}^{2}}+\sqrt{t^{2}+\beta_{2}^{2}}}\to 0$$

as $t\to \infty ,\Delta \left(t\right)\to \Delta \left(\infty \right)=\alpha_{1}-\alpha_{2}$ as t→∞. As

$$\Delta^{\prime }\left(t\right)=\frac{t}{\sqrt{t^{2}+\beta_{1}^{2}}}-\frac{t}{\sqrt{t^{2}+\beta_{2}^{2}}}\text{,}$$

if $\beta_{1}<\beta_{2}$ we have $\Delta^{\prime }>0$ and hence Δ>0 iff Δ(0)≥0, and Δ<0 iff Δ(∞)≤0. As $\min\left(\alpha_{1}+\beta_{1},\alpha_{2}+\beta_{2}\right)\geq 0,G_{\alpha_{1},\beta_{1}}<G_{\alpha_{2},\beta_{2}}$ (or >) iff Δ>0 (or <). Otherwise, i.e., iff $\alpha_{1}>\alpha_{2}$ and $\alpha_{1}+\beta_{1}<\alpha_{2}+\beta_{2},\Delta$ has a unique zero $t^{*}$ in (0,∞), which can be determined as follows. Let $u=\sqrt{t^{2}+\beta_{1}^{2}}>\beta_{1}$ so that $t=\sqrt{u^{2}-\beta_{1}^{2}}$ and $t^{2}+\beta_{2}^{2}=u^{2}+\left(\beta_{2}^{2}-\beta_{1}^{2}\right)$, and Δ(t)=0 iff

$$\alpha_{1}+u-\alpha_{2}=\sqrt{u^{2}+\left(\beta_{2}^{2}-\beta_{1}^{2}\right)}\text{.}$$

Taking squares,

$$u^{2}+2\left(\alpha_{1}-\alpha_{2}\right)u+{\left(\alpha_{1}-\alpha_{2}\right)}^{2}=u^{2}+\left(\beta_{2}^{2}-\beta_{1}^{2}\right)$$

from which

$$u=\frac{\beta_{2}^{2}-\beta_{1}^{2}}{2\left(\alpha_{1}-\alpha_{2}\right)}-\frac{\alpha_{1}-\alpha_{2}}{2}\text{.}$$

Then

$$u-\beta_{1}=\frac{\left(\beta_{2}^{2}-\beta_{1}^{2}\right)-{\left(\alpha_{1}-\alpha_{2}\right)}^{2}}{2\left(\alpha_{1}-\alpha_{2}\right)}-\beta_{1}=\frac{\left(\beta_{2}-\beta_{1}-\alpha_{1}+\alpha_{2}\right)\left(\beta_{2}+\beta_{1}+\alpha_{1}-\alpha_{2}\right)}{2\left(\alpha_{1}-\alpha_{2}\right)}\text{.}$$

The numerator equals $\left(\left(\alpha_{2}+\beta_{2}\right)-\left(\alpha_{1}+\beta_{1}\right)\right)\left(\left(\alpha_{1}-\alpha_{2}\right)+\left(\beta_{1}+\beta_{2}\right)\right)>0$ so that indeed $u>\beta_{1}$. Similarly,

$$u+\beta_{1}=\frac{\beta_{2}^{2}-\beta_{1}^{2}+2\beta_{1}\left(\alpha_{1}-\alpha_{2}\right)-{\left(\alpha_{1}-\alpha_{2}\right)}^{2}}{2\left(\alpha_{1}-\alpha_{2}\right)}=\frac{\left(\beta_{2}+\beta_{1}-\alpha_{1}+\alpha_{2}\right)\left(\beta_{2}-\beta_{1}+\alpha_{1}-\alpha_{2}\right)}{2\left(\alpha_{1}-\alpha_{2}\right)}$$

so that with $t^{2}=u^{2}-\beta_{1}^{2}=\left(u-\beta_{1}\right)\left(u+\beta_{1}\right)$ we indeed obtain

$$t=\frac{\sqrt{\left({\left(\beta_{2}-\beta_{1}\right)}^{2}-{\left(\alpha_{1}-\alpha_{2}\right)}^{2}\right)\left({\left(\beta_{2}+\beta_{1}\right)}^{2}-{\left(\alpha_{1}-\alpha_{2}\right)}^{2}\right)}}{2\left(\alpha_{1}-\alpha_{2}\right)}$$

for the unique solution of Δ(t)=0 (and equivalently $G_{\alpha_{1},\beta_{1}}\left(t\right)=G_{\alpha_{2},\beta_{2}}\left(t\right)$) on (0,∞). Clearly, Δ(s)<0 for 0≤s<t and Δ(s)>0 for s>t, so that $G_{\alpha_{1},\beta_{1}}\left(s\right)>G_{\alpha_{2},\beta_{2}}\left(s\right)$ for 0<s<t and $G_{\alpha_{1},\beta_{1}}\left(s\right)<G_{\alpha_{2},\beta_{2}}\left(s\right)$ for s>t, and the proof is complete. □

Lemma 4 *Suppose the quadratic polynomial* $Q\left(t\right)=t^{2}+\gamma t+\delta$ *has two real zeros* $t_{1}\leq t_{2}$ *. Then* Q(t)<0 *iff* $t_{1}<t<t_{2}$*.*

Proof Trivial, as $Q\left(t\right)=\left(t-t_{1}\right)\left(t-t_{2}\right)$. □

## 3 Previous work

Amos [1] gives the bounds

$$G_{\nu +1/2,\nu +3/2}\leq R_{\nu }\leq G_{\nu +1/2,\nu +1/2}\text{,}\;\nu \geq 0$$

(Eq. (16)) and

$$G_{\nu +1,\nu +1}\leq R_{\nu }\leq G_{\nu ,\nu +2}\leq G_{\nu ,\nu }\text{,}\;\nu \geq 0$$

(Eqs. (9) and (11)). Using Lemma 3 with $\beta_{1}=\nu +1<\nu +3/2=\beta_{2}$ and $\alpha_{1}+\beta_{1}=2\nu +2=\alpha_{2}+\beta_{2}$ we see that the first lower bound is uniformly better (larger) than the second one, whereas again with Lemma 3, neither of the upper bounds $G_{\nu +1/2,\nu +1/2}$ and $G_{\nu ,\nu +2}$ is uniformly better (smaller) than the other: in fact, with $\alpha_{1}-\alpha_{2}=1/2,\beta_{2}-\beta_{1}=3/2$ and $\beta_{2}+\beta_{1}=2\nu +5/2$, we get

$$t=\frac{\sqrt{\left(9/4-1/4\right)\left(4\nu^{2}+10\nu +25/4-1/4\right)}}{2\cdot \left(1/2\right)}=2\sqrt{\left(\nu +1\right)\left(2\nu +3\right)}\text{,}$$

so that $G_{\nu ,\nu +2}\left(s\right)<G_{\nu +1/2,\nu +1/2}\left(s\right)$ for 0<s<t and $G_{\nu +1/2,\nu +1/2}\left(s\right)<G_{\nu ,\nu +2}\left(s\right)$ for s>t.

Nåsell [5] gives rational bounds for $R_{\nu }$, and notes (p. 8) that the Amos-type bounds $G_{\nu +1/2,\nu +3/2}<R_{\nu }$ and $R_{\nu }<G_{\nu +1/2,\nu +1/2}$ are valid for ν>−1 and ν>−1/2, respectively. But trivially $R_{-1/2}=\tanh<1=G_{0,0}$, so that the upper bound is in fact valid for ν≥−1/2.

Simpson and Spector [9, Theorem 2] show that

$$v_{\nu }{\left(t\right)}^{2}-\left(2\nu +1\right)v_{\nu }\left(t\right)-\left(t^{2}+\nu +1/2\right)>0\text{,}\;t>0,\;\nu \geq 0\text{.}$$

As the quadratic function $Q\left(s\right)=s^{2}-\left(2\nu +1\right)s-\left(t^{2}+\nu +1/2\right)$ has zeros

$$\nu +1/2\pm \sqrt{{\left(\nu +1/2\right)}^{2}+\left(t^{2}+\nu +1/2\right)}=\nu +1/2\pm \sqrt{t^{2}+\left(\nu +1/2\right)\left(\nu +3/2\right)}\text{,}$$

Lemma 4 implies that $v_{\nu }\left(t\right)>\nu +1/2+\sqrt{t^{2}+\left(\nu +1/2\right)\left(\nu +3/2\right)}$ and hence

$$R_{\nu }<G_{\nu +1/2,\sqrt{\left(\nu +1/2\right)\left(\nu +3/2\right)}}\text{,}\;\nu \geq 0\text{.}$$

Using Lemma 3, we see that this bound is uniformly better than the Amos-type bound $G_{\nu +1/2,\nu +1/2}$. To compare with $G_{\nu ,\nu +2}$, note that

$$\left({\left(\beta_{2}-\beta_{1}\right)}^{2}-{\left(\alpha_{1}-\alpha_{2}\right)}^{2}\right)\left({\left(\beta_{2}+\beta_{1}\right)}^{2}-{\left(\alpha_{1}-\alpha_{2}\right)}^{2}\right)={\left(\beta_{2}^{2}-\beta_{1}^{2}\right)}^{2}-2\left(\beta_{2}^{2}+\beta_{1}^{2}\right){\left(\alpha_{1}-\alpha_{2}\right)}^{2}+{\left(\alpha_{1}-\alpha_{2}\right)}^{4}\text{.}$$

Thus, using Lemma 3 with $\alpha_{1}=\nu +1/2,\beta_{1}=\sqrt{\left(\nu +1/2\right)\left(\nu +3/2\right)},\alpha_{2}=\nu$ and $\beta_{2}=\nu +2$, we get $\alpha_{1}-\alpha_{2}=1/2,\beta_{2}^{2}-\beta_{1}^{2}=2\nu +13/4,\beta_{2}^{2}+\beta_{1}^{2}=2\nu^{2}+6\nu +19/4$ and

$$t=\sqrt{{\left(2\nu +13/4\right)}^{2}-2\left(2\nu^{2}+6\nu +19/4\right)/4+1/16}=\sqrt{3\nu^{2}+10\nu +33/4}=\sqrt{\left(3\nu +11/2\right)\left(\nu +3/2\right)}\text{,}$$

and therefore $G_{\nu +1/2,\sqrt{\left(\nu +1/2\right)\left(\nu +3/2\right)}}\left(s\right)<G_{\nu ,\nu +2}\left(s\right)$ for s>t, and $G_{\nu ,\nu +2}\left(s\right)<G_{\nu +1/2,\sqrt{\left(\nu +1/2\right)\left(\nu +3/2\right)}}\left(s\right)$ for 0<s<t.

Neuman [6, Proposition 5] shows that

$$v_{\nu }^{2}\left(t\right)-\left(2\nu +1\right)v_{\nu }\left(t\right)-\left(t^{2}+\nu +1/2\right)<\nu +3/2\text{,}\;t>0,\;\nu >-3/2\text{.}$$

As the quadratic function $Q\left(s\right)=s^{2}-\left(2\nu +1\right)s-\left(t^{2}+2\left(\nu +1\right)\right)$ has zeros

$$\nu +1/2\pm \sqrt{{\left(\nu +1/2\right)}^{2}+t^{2}+2\left(\nu +1\right)}=\nu +1/2\pm \sqrt{t^{2}+{\left(\nu +3/2\right)}^{2}}\text{,}$$

Lemma 4 implies that $v_{\nu }\left(t\right)<\nu +1/2+\sqrt{t^{2}+{\left(\nu +3/2\right)}^{2}}$ for t>0 and ν>−3/2. If $\nu \geq -1,v_{\nu }>0$ and hence $R_{\nu }>G_{\nu +1/2,\nu +3/2}$.

Yuan and Kalbfleisch [11, Eq. (A.5)] show that

$$G_{\nu +1,\nu +1}\leq R_{\nu }\leq G_{\nu ,\nu }\text{,}\;\nu >-1\text{.}$$

Baricz and Neuman [2, Theorems 2.1 and 2.2] show that if a>1 and b=1/(4log(a)), then

$$v_{\nu }{\left(t\right)}^{2}-\left(2\nu +1\right)v_{\nu }\left(t\right)-t^{2}<2\left(\nu +1\right)\text{,}\;0<t\leq 2b,\;\nu \geq b-2$$

and that

$$v_{\nu }{\left(t\right)}^{2}-2\nu v_{\nu }\left(t\right)-t^{2}>4\left(\nu +1\right)\text{,}\;t>0,\;\nu >-2$$

(the reference uses p−1 for ν). The former extends the earlier result of Neuman  [6] when ν≤−3/2, in which case the bounds are not valid for all t>0. As $s\mapsto Q\left(s\right)=s^{2}-2\nu s-\left(t^{2}+4\left(\nu +1\right)\right)$ has zeros

$$\nu \pm \sqrt{\nu^{2}+t^{2}+4\left(\nu +1\right)}=\nu \pm \sqrt{t^{2}+{\left(\nu +2\right)}^{2}}\text{,}$$

Lemma 4 yields that for ν≥−1, the latter is equivalent to $R_{\nu }<G_{\nu ,\nu +2}$, extending the previously established ν range for this bound.

Laforgia and Natalini [4, Theorem 1.1] show that

$$\frac{-\nu +\sqrt{t^{2}+\nu^{2}}}{t}<\frac{I_{\nu }\left(t\right)}{I_{\nu -1}\left(t\right)}\text{,}\;t>0,\;\nu \geq 0$$

(the condition that t>0 is not stated explicitly in the theorem, but given in Eq. (1.8) of the reference used in the proof). As

$$\frac{\sqrt{t^{2}+\nu^{2}}-\nu }{t}=\frac{\left(t^{2}+\nu^{2}\right)-\nu^{2}}{t\left(\sqrt{t^{2}+\nu^{2}}+\nu \right)}=\frac{t}{\nu +\sqrt{t^{2}+\nu^{2}}}=G_{\nu ,\nu }\left(t\right)\text{,}$$

the result is equivalent to

$$R_{\nu }>G_{\nu +1,\nu +1}\text{,}\;\nu \geq -1\text{,}$$

which is weaker than the $R_{\nu }>G_{\nu +1/2,\nu +3/2}$ bound.

Segura [8, Theorem 3] shows that

$$\frac{I_{\nu +1/2}\left(t\right)}{I_{\nu -1/2}\left(t\right)}<\frac{t}{\nu +\sqrt{t^{2}+\nu^{2}}}\text{,}\;t>0,\;\nu \geq 0$$

or equivalently, $R_{\nu }<G_{\nu +1/2,\nu +1/2}$ for ν≥−1/2. For $r_{\nu }\left(t\right)=I_{\nu }\left(t\right)/\left(tI_{\nu -1}\left(t\right)\right)=R_{\nu -1}\left(t\right)/t$, Segura [8, Eqs. (22) and (61)] also shows that for t>0 and ν≥0,

$$\frac{1}{\left(\nu -1/2\right)+\sqrt{t^{2}+{\left(\nu +1/2\right)}^{2}}}<r_{\nu }\left(t\right)<\frac{1}{\nu +\sqrt{\nu^{2}+t^{2}\nu /\left(\nu +1\right)}}\text{.}$$

Clearly, the lower bound is equivalent to $R_{\nu }>G_{\nu +1/2,\nu +3/2}$ for ν≥−1, and the upper bound to

$$R_{\nu }\left(t\right)<\frac{t}{\nu +1+\sqrt{{\left(\nu +1\right)}^{2}+t^{2}\left(\nu +1\right)/\left(\nu +2\right)}}$$

for t>0 and ν≥−1, which is weaker than the upper bound $R_{\nu }<G_{\nu ,\nu +2}$.

Kokologiannaki [3, Theorem 2.1] shows that for $f_{\nu }\left(t\right)=I_{\nu +1}\left(t\right)/\left(tI_{\nu }\left(t\right)\right)=R_{\nu }\left(t\right)/t$,

$$-\frac{\nu +1}{t^{2}}+\sqrt{\frac{{\left(\nu +1\right)}^{2}}{t^{4}}+\frac{1}{t^{2}}}<f_{\nu }\left(t\right)<-\frac{\nu +1}{t^{2}}+\sqrt{\frac{{\left(\nu +1\right)}^{2}}{t^{4}}+\frac{1}{t^{2}}+\frac{1}{4{\left(\nu +1\right)}^{2}\left(\nu +2\right)}}$$

for t>0 and ν>−1. As

$$-\frac{\nu +1}{t}+\sqrt{\frac{{\left(\nu +1\right)}^{2}}{t^{2}}+1}=\frac{\sqrt{t^{2}+{\left(\nu +1\right)}^{2}}-\left(\nu +1\right)}{t}\text{,}$$

the lower bound again is equivalent to $R_{\nu }>G_{\nu +1,\nu +1}$ for ν>−1. Write $U_{K}\left(t\right)$ for the above upper bound and $\gamma =1/\left(4{\left(\nu +1\right)}^{2}\left(\nu +2\right)\right)$. $U_{K}\left(t\right)$ is the larger root of the quadratic polynomial

$$s\mapsto Q\left(s;t\right)=s^{2}+\frac{2\left(\nu +1\right)}{t^{2}}s-\frac{1}{t^{2}}-\gamma \text{,}$$

so by Lemma 4, for any function s(t) with Q(s(t),t)<0 for all t>0 we have $s<U_{K}$. Consider $s\left(t\right)=G_{\nu ,\nu +2}\left(t\right)/t$, and write β=ν+2. Then Q(s(t),t)<0 iff

$$\frac{1}{{\left(\nu +\sqrt{t^{2}+\beta^{2}}\right)}^{2}}+\frac{2\left(\nu +1\right)}{t^{2}}\frac{1}{\nu +\sqrt{t^{2}+\beta^{2}}}<\frac{1}{t^{2}}+\gamma \text{,}$$

which in turn is equivalent to

$$\left(1+\gamma t^{2}\right){\left(\nu +\sqrt{t^{2}+\beta^{2}}\right)}^{2}-2\left(\nu +1\right)\left(\nu +\sqrt{t^{2}+\beta^{2}}\right)-t^{2}>0\text{.}$$

Let $\xi =\sqrt{t^{2}+\beta^{2}}-\beta$ so that t≠0 iff $\xi >0,t^{2}={\left(\xi +\beta \right)}^{2}-\beta^{2}=\xi \left(\xi +2\beta \right),\nu +\sqrt{t^{2}+\beta^{2}}=2\left(\nu +1\right)+\xi$, and the inequality becomes

$$0<P\left(\xi \right)=\gamma \xi^{4}+\gamma \left(4\left(\nu +1\right)+2\beta \right)\xi^{3}+\left(1+8\left(\nu +1\right)\beta \gamma +4{\left(\nu +1\right)}^{2}\gamma -1\right)\xi^{2}\;+\left(4\left(\nu +1\right)+8{\left(\nu +1\right)}^{2}\beta \gamma -2\left(\nu +1\right)-2\beta \right)\xi +\left(4{\left(\nu +1\right)}^{2}-4{\left(\nu +1\right)}^{2}\right)\text{.}$$

The coefficient of the linear term is 0, so that

$$P\left(\xi \right)=\gamma \xi^{2}\left(\xi^{2}+\left(4\left(\nu +1\right)+2\beta \right)\xi +\left(8\left(\nu +1\right)\beta +4{\left(\nu +1\right)}^{2}\right)\right)$$

and for ν>−1 we have P(ξ)>0 for ξ>0. Thus, $G_{\nu ,\nu +2}\left(t\right)/t<U_{K}\left(t\right)$ for all t>0. We thus have the following.

Theorem 2 *For all* t>0 *and* ν>−1*,*

$$\frac{G_{\nu ,\nu +2}\left(t\right)}{t}<-\frac{\nu +1}{t^{2}}+\sqrt{\frac{{\left(\nu +1\right)}^{2}}{t^{4}}+\frac{1}{t^{2}}+\frac{1}{4{\left(\nu +1\right)}^{2}\left(\nu +2\right)}}\text{.}$$

Hence, the upper bound in Kokologiannaki  [3, Theorem 2.1] is strictly weaker than the bound $f_{\nu }\left(t\right)=R_{\nu }\left(t\right)/t<G_{\nu ,\nu +2}\left(t\right)/t$.

The various results can be summarized as follows: the “best” (in the sense of not being uniformly weaker than other) Amos-type bounds for $R_{\nu }$ currently available are

$$G_{\nu +1/2,\nu +3/2}<R_{\nu }\text{,}\;\nu \geq -1\text{,}$$

$$R_{\nu }<G_{\nu ,\nu +2}\text{,}\;\nu \geq -1\text{,}$$

$$R_{\nu }<G_{\nu +1/2,\sqrt{\left(\nu +1/2\right)\left(\nu +3/2\right)}}\text{,}\;\nu >0\text{,}$$

$$R_{\nu }<G_{\nu +1/2,\nu +1/2}\text{,}\;-1/2\leq \nu \leq 0\text{.}$$

## 4 Results

Theorem 3 *For* ν≥−1*,*

$$L_{\nu }=\left\{\left(\alpha ,\beta \right):\alpha \geq \nu +1/2,\alpha +\beta \geq 2\left(\nu +1\right),\beta \geq 0\right\}$$

*and* $G_{\nu +1/2,\nu +3/2}$ *is the maximum of the family* $G_{L_{\nu }}$ *of lower Amos-type bounds for* $R_{\nu }$*.*

Proof We already know that for $\nu \geq -1,G_{\nu +1/2,\nu +3/2}<R_{\nu }$. By Theorem 1, $G_{\alpha ,\beta }\leq R_{\nu }$ is only possible if α+β≥2(ν+1)=(ν+1/2)+(ν+3/2) and α≥ν+1/2. If β<ν+3/2, Lemma 3 implies that $G_{\alpha ,\beta }<G_{\nu +1/2,\nu +3/2}$. Otherwise, we trivially have $G_{\alpha ,\beta }\leq G_{\alpha ,\nu +3/2}\leq G_{\nu +1/2,\nu +3/2}$. □

Theorem 4 *For* $\nu \geq -1,U_{\nu }$ *is a closed convex set.*

Proof For fixed $t>0,\left(\alpha ,\beta \right)\mapsto h_{\alpha ,\beta }\left(t\right)$ is continuous, linear in α, and satisfies $\partial h_{\alpha ,\beta }\left(t\right)/\partial \beta =\beta {\left(t^{2}+\beta^{2}\right)}^{-1/2}\geq 0$ and hence

$$\frac{\partial^{2}h_{\alpha ,\beta }\left(t\right)}{\partial \beta^{2}}={\left(t^{2}+\beta^{2}\right)}^{-1/2}-\beta^{2}{\left(t^{2}+\beta^{2}\right)}^{-3/2}=t^{2}{\left(t^{2}+\beta^{2}\right)}^{-3/2}\geq 0$$

and is thus convex. By Theorem 1, $G_{\alpha ,\beta }\geq R_{\nu }$ is only possible when α+β≥0, for which it is equivalent to $h_{\alpha ,\beta }\leq v_{\nu }$. Hence,

$$U_{\nu }={⋂}_{t>0}\left\{\left(\alpha ,\beta \right):h_{\alpha ,\beta }\left(t\right)\leq v\left(t\right)\right\}$$

is the intersection of closed convex sets, and thus a closed convex set. □

Let

$$V_{\nu }\left(\alpha \right)=\left\{\beta :\left(\alpha ,\beta \right)\in U_{\nu }\right\}$$

$$\beta_{\nu }^{*}\left(\alpha \right)=\sup V_{\nu }\left(\alpha \right)$$

$$\alpha_{\nu }^{*}=\sup\left\{\alpha :V_{\nu }\left(\alpha \right)\neq 0̸\right\}\text{.}$$

As $\lim_{\beta \to \infty }G_{\alpha ,\beta }\left(t\right)=0$ for t>0, clearly $\beta_{\nu }^{*}\left(\alpha \right)<\infty$ for ν≥−1.

Theorem 5 *For* ν≥−1*,*

$$U_{\nu }=\left\{\left(\alpha ,\beta \right):\alpha \leq \alpha_{\nu }^{*},\max\left(0,-\alpha \right)\leq \beta \leq \overset{*}{\underset{\nu }{\beta }}\left(\alpha \right)\right\}\text{,}$$

*with* $\beta_{\nu }^{*}$ *continuous, decreasing and concave.*

Proof For ν≥−1, we have $\beta \in V_{\nu }\left(\alpha \right)$ iff α+β≥0 and $h_{\alpha ,\beta }\leq v_{\nu }$. Thus, as $h_{\alpha ,\beta }$ is continuous and increasing in β, if $V_{\nu }\left(\alpha \right)$ is non-empty, it is the closed interval $\left[\max\left(0,-\alpha \right),\beta_{\nu }^{*}\left(\alpha \right)\right]$. By Lemma 3, $G_{\alpha -\eta ,\beta +\eta }>G_{\alpha ,\beta }$ for all η>0, so $\beta_{\nu }^{*}$ must be decreasing as long as $V_{\nu }\left(\alpha \right)$ is non-empty. If $\alpha_{n}↑\alpha_{\nu }^{*},\beta_{n}=\beta_{\nu }^{*}\left(\alpha_{n}\right)$ is decreasing and non-negative and thus must have a finite limit $\beta_{\infty }$. Taking limits in $\alpha_{n}+\beta_{n}\geq 0$ and $h_{\alpha_{n},\beta_{n}}\leq v_{\nu }$ implies that $\alpha_{\nu }^{*}+\beta_{\infty }\geq 0$ and $h_{\alpha_{\nu }^{*},\beta_{\infty }}\leq v_{\nu }$. Thus, $V_{\nu }\left(\alpha_{\nu }^{*}\right)$ is non-empty. As $U_{\nu }={⋃}_{\alpha }V_{\nu }\left(\alpha \right)$, the first assertion follows. Finally, as $U_{\nu }$ is closed and convex, $\beta_{\nu }^{*}$ must be continuous and concave. □

Theorem 6 *Let* ν≥−1 *. For* $\alpha \leq \nu ,\beta_{\nu }^{*}\left(\alpha \right)=2\left(\nu +1\right)-\alpha$ *. For* $\nu <\alpha \leq \alpha_{\nu }^{*},\beta_{\nu }^{*}\left(\alpha \right)<2\left(\nu +1\right)-\alpha$*.*

Proof We know that $\left(\nu ,\nu +2\right)\in U_{\nu }$. By Theorem 1, $G_{\alpha ,\beta }\geq R_{\nu }$ is only possible if α+β≤2(ν+1)=ν+(ν+2) so that $\beta_{\nu }^{*}\left(\alpha \right)\leq 2\left(\nu +1\right)-\alpha$. If α+β=2(ν+1) and β>0,

$$h_{\alpha ,\beta }\left(t\right)=2\left(\nu +1\right)+\frac{t^{2}}{2\beta }+O\left(t^{4}\right)\text{,}\;t\to 0$$

by Eq. (3) and comparison with Eq. (2) shows that $h_{\alpha ,\beta }\leq v_{\nu }$ is only possible if in fact β≥ν+2>0, or equivalently, if α≤2(ν+1)−(ν+2)=ν. For α<ν, Lemma 3 implies that $G_{\alpha ,2\left(\nu +1\right)-\alpha }>G_{\nu ,\nu +2}\geq R_{\nu }$, so that indeed $\beta_{\nu }^{*}\left(\alpha \right)=2\left(\nu +1\right)-\alpha$. □

Let

$$Q_{\alpha ,\beta }\left(s\right)=\beta^{2}+\left(2\left(\nu +1\right)\alpha -\alpha^{2}-\beta^{2}\right)s+2\left(\nu +1/2-\alpha \right)s^{2}\text{.}$$

Lemma 5 *Let* $\Delta =v_{\nu }-h_{\alpha ,\beta }$ *. Then*

$$t\Delta^{\prime }\left(t\right)=\frac{Q_{\alpha ,\beta }\left(\sqrt{t^{2}+\beta^{2}}\right)}{\sqrt{t^{2}+\beta^{2}}}+\left(2\left(\nu +1\right)-v_{\nu }\left(t\right)-h_{\alpha ,\beta }\left(t\right)\right)\Delta \left(t\right)\text{.}$$

Proof As shown in Simpson and Spector [9], $v_{\nu }$ satisfies the Riccati equation $tv_{\nu }^{\prime }\left(t\right)=t^{2}+2\left(\nu +1\right)v_{\nu }\left(t\right)-v_{\nu }{\left(t\right)}^{2}$ and clearly, $h_{\alpha ,\beta }^{\prime }\left(t\right)=t/\sqrt{t^{2}+\beta^{2}}$. Hence, as $v^{2}=h^{2}+\left(v^{2}-h^{2}\right)=h^{2}+\left(v-h\right)\left(v+h\right)$,

$$tv_{\nu }^{\prime }\left(t\right)=t^{2}+2\left(\nu +1\right)\left(\Delta \left(t\right)+h_{\alpha ,\beta }\left(t\right)\right)-\left(h_{\alpha ,\beta }{\left(t\right)}^{2}+\Delta \left(t\right)\left(v_{\nu }\left(t\right)+h_{\alpha ,\beta }\left(t\right)\right)\right)=t^{2}+2\left(\nu +1\right)h_{\alpha ,\beta }\left(t\right)-h_{\alpha ,\beta }{\left(t\right)}^{2}+\left(2\left(\nu +1\right)-v_{\nu }\left(t\right)-h_{\alpha ,\beta }\left(t\right)\right)\Delta \left(t\right)$$

with

$$t^{2}+2\left(\nu +1\right)h_{\alpha ,\beta }\left(t\right)-h_{\alpha ,\beta }{\left(t\right)}^{2}=t^{2}+2\left(\nu +1\right)\left(\alpha +\sqrt{t^{2}+\beta^{2}}\right)-\left(\alpha^{2}+2\alpha \sqrt{t^{2}+\beta^{2}}+t^{2}+\beta^{2}\right)=2\left(\nu +1\right)\alpha -\alpha^{2}-\beta^{2}+2\left(\nu +1-\alpha \right)\sqrt{t^{2}+\beta^{2}}$$

so that

$$t^{2}+2\left(\nu +1\right)h_{\alpha ,\beta }\left(t\right)-h_{\alpha ,\beta }{\left(t\right)}^{2}-\frac{t^{2}}{\sqrt{t^{2}+\beta^{2}}}=\frac{\left(2\left(\nu +1\right)\alpha -\alpha^{2}-\beta^{2}\right)\sqrt{t^{2}+\beta^{2}}+2\left(\nu +1-\alpha \right)\left(t^{2}+\beta^{2}\right)-t^{2}}{\sqrt{t^{2}+\beta^{2}}}=\frac{Q_{\alpha ,\beta }\left(\sqrt{t^{2}+\beta^{2}}\right)}{\sqrt{t^{2}+\beta^{2}}}\text{,}$$

whence the lemma. □

Let

$$\alpha_{\nu }^{♭}=\min\left(\nu +1/2,2\nu +1\right)$$

(so that $\alpha_{\nu }^{♭}$ equals ν+1/2 for ν≥−1/2 and 2ν+1 otherwise), and for $-1\leq \nu \leq \alpha \leq \alpha_{\nu }^{♭}$ let

$$\beta_{\nu }^{♭}\left(\alpha \right)=\sqrt{2\nu +1-2\alpha }+\sqrt{2\nu +1+2\nu \alpha -\alpha^{2}}=\sqrt{2\left(\nu +1/2-\alpha \right)}+\sqrt{\left(\alpha +1\right)\left(2\nu +1-\alpha \right)}$$

(where the second expressions shows that $\beta_{\nu }^{♭}$ is well-defined).

Lemma 6 *Let* ν≥−1 *. Then* $\beta_{\nu }^{♭}$ *is strictly concave with* $\beta_{\nu }^{♭}\left(\nu \right)=\nu +2,\beta_{\nu }^{♭}\left(\alpha_{\nu }^{♭}\right)$ *equals* $\sqrt{\left(\nu +1/2\right)\left(\nu +3/2\right)}$ *if* ν≥−1/2 *and* $\sqrt{-2\left(\nu +1/2\right)}$ *if* −1≤ν≤−1/2*, and* $\alpha \mapsto \alpha +\beta_{\nu }^{♭}\left(\alpha \right)$ *is non-negative and decreasing.*

Proof The assertions about the values of $\beta_{\nu }^{♭}$ at ν and $\alpha_{\nu }^{♭}$ are straightforward. If $\nu =-1,\alpha_{\nu }^{♭}=\nu$ and there is nothing left to prove. Hence, take ν>−1. The second derivative of $\alpha \mapsto \sqrt{f\left(\alpha \right)}$ is given by

$$\frac{d^{2}\sqrt{f\left(\alpha \right)}}{d\alpha^{2}}=\frac{f^{\prime \prime }\left(\alpha \right)f\left(\alpha \right)-f^{\prime }{\left(\alpha \right)}^{2}/2}{2{\sqrt{f\left(\alpha \right)}}^{3}}\text{.}$$

For $f_{1}\left(\alpha \right)=2\left(\nu +1/2-\alpha \right)$ and $f_{2}\left(\alpha \right)=2\nu +1+2\nu \alpha -\alpha^{2}$ we have $f_{1}^{\prime }\left(\alpha \right)=-2,f_{1}^{\prime \prime }\left(\alpha \right)=0,f_{2}^{\prime }\left(\alpha \right)=2\left(\nu -\alpha \right)$ and $f_{2}^{\prime \prime }\left(\alpha \right)=-2$, giving numerators −2 and $-2\left(2\nu +1+2\nu \alpha -\alpha^{2}\right)-4{\left(\nu -\alpha \right)}^{2}/2=-2{\left(\nu +1\right)}^{2}<0$. Hence $\beta_{\nu }^{♭}$ is the sum of two strictly concave functions, and thus strictly concave. Clearly,

$$\frac{d\beta_{\nu }^{♭}\left(\alpha \right)}{d\alpha }=\frac{-1}{\sqrt{2\nu +1-2\alpha }}+\frac{\nu -\alpha }{\sqrt{2\nu +1+2\nu \alpha -\alpha^{2}}}$$

with value −1 at α=ν. By strict concavity, the derivative of $\beta_{\nu }^{♭}$ is decreasing, and hence less than −1 for α>ν, so that the derivative of $\alpha \mapsto f\left(\alpha \right)=\alpha +\beta_{\nu }^{♭}\left(\alpha \right)$ is negative for α>ν and f is decreasing. It remains to show that $f\left(\alpha_{\nu }^{♭}\right)\geq 0$. If ν≥−1/2, this is immediate from $\alpha_{\nu }^{♭}=\nu +1/2\geq 0$. Otherwise, $\alpha_{\nu }^{♭}=2\nu +1<0$ and $f\left(\alpha_{\nu }^{♭}\right)=2\nu +1+\sqrt{-\left(2\nu +1\right)}$, which is non-negative as 0≤−(2ν+1)≤1. □

Theorem 7 *Let* ν≥−1 *. Then for* $\nu \leq \alpha \leq \alpha_{\nu }^{♭},G_{\alpha ,\beta_{\nu }^{♭}\left(\alpha \right)}\geq R_{\nu }$*.*

Proof The proof will be based on the ideas of Simpson and Spector [9]. Suppose Δ is sufficiently often continuously differentiable on [0,∞) with Δ(0)>0. Suppose that for all t>0,Δ(t)=0 implies that there exists a suitable odd k such that $\Delta^{\left(l\right)}\left(t\right)=0$ for l<k and $\Delta^{\left(k\right)}\left(t\right)>0$. Then Δ(t)≥0 for all t≥0, as otherwise for s=inf{t>0:Δ(t)=0} we would have $\Delta \left(s-\epsilon \right)=\Delta^{\left(k\right)}\left(s^{*}\right){\left(-\epsilon \right)}^{k}/k!<0$ for all sufficiently small ϵ>0 and a suitable $s^{*}\in \left(s-\epsilon ,s\right)$, which is impossible.

In our case, $\Delta =v_{\nu }-h_{\alpha ,\beta }$, where $\beta =\beta_{\nu }^{♭}\left(\alpha \right)$. If α=ν, we have β=ν+2 and we already know for ν≥−1 that $G_{\alpha ,\beta }=G_{\nu ,\nu +2}\geq R_{\nu }$. By Lemma 6, $\alpha +\beta_{\nu }^{♭}\left(\alpha \right)$ is decreasing and hence maximal for α=ν with value 2(ν+1). Thus, for α>ν we have $\alpha +\beta_{\nu }^{♭}\left(\alpha \right)<2\left(\nu +1\right)$, or equivalently, Δ(0)>0.

Write $s\left(t\right)=\sqrt{t^{2}+\beta^{2}}$. If α=ν+1/2, which is only possible if ν≥−1/2, we have $\beta =\sqrt{\left(\nu +1/2\right)\left(\nu +3/2\right)}$ and $Q_{\alpha ,\beta }=\beta^{2}$ for all s. If ν=−1/2, we already know that $R_{-1/2}=\tanh\leq G_{0,0}$. Otherwise, $Q_{\alpha ,\beta }\left(s\right)=\beta^{2}>0$. If Δ(t)=0 for some t>0, Lemma 5 implies that $\Delta^{\prime }\left(t\right)=\beta^{2}/\left(ts\left(t\right)\right)>0$, completing the proof for this case.

Hence, consider the case where ν<α<ν+1/2. Solving $Q_{\alpha ,\beta }\left(s\right)=0$ has discriminant

$${\left(2\left(\nu +1\right)\alpha -\alpha^{2}-\beta^{2}\right)}^{2}-8\left(\nu +1/2-\alpha \right)\beta^{2}=\left(2\left(\nu +1\right)\alpha -\alpha^{2}-\beta^{2}+2\beta \sqrt{2\nu +1-2\alpha }\right)\cdot \left(2\left(\nu +1\right)\alpha -\alpha^{2}-\beta^{2}-2\beta \sqrt{2\nu +1-2\alpha }\right)\text{,}$$

with $\beta =\beta_{\nu }^{♭}\left(\alpha \right)$ the larger root of the first factor. Hence, the discriminant vanishes, and with

$$\sigma =-\frac{2\left(\nu +1\right)\alpha -\alpha^{2}-\beta^{2}}{4\left(\nu +1/2-\alpha \right)}=\frac{2\sqrt{2\nu +1-2\alpha }\beta }{4\left(\nu +1/2-\alpha \right)}=\frac{\beta }{\sqrt{2\nu +1-2\alpha }}>0$$

we have $Q_{\alpha ,\beta }\left(s\right)=\gamma {\left(s-\sigma \right)}^{2}$, where γ=2ν+1−2α>0.

If Δ(t)=0 for some t>0, Lemma 5 implies that $t\Delta^{\prime }\left(t\right)=Q_{\alpha ,\beta }\left(s\left(t\right)\right)/s\left(t\right)$. If $s\left(t\right)\neq \sigma ,Q_{\alpha ,\beta }\left(s\left(t\right)\right)>0$, and the proof is complete. Otherwise, use Lemma 5 to write $t\Delta^{\prime }\left(t\right)=\xi \left(t\right)+\eta \left(t\right)\Delta \left(t\right)$, where

$$\xi \left(t\right)=\gamma {\left(s\left(t\right)-\sigma \right)}^{2}/s\left(t\right)=\gamma \left(s\left(t\right)-2\sigma +\frac{\sigma^{2}}{s\left(t\right)}\right)$$

so that $\xi^{\prime }\left(t\right)=\gamma \left(s^{\prime }\left(t\right)-\sigma^{2}s^{\prime }\left(t\right)/s{\left(t\right)}^{2}\right)$ and

$$\xi^{\prime \prime }\left(t\right)=\gamma \left(s^{\prime \prime }\left(t\right)-\sigma^{2}\left(\frac{s^{\prime \prime }\left(t\right)}{s{\left(t\right)}^{2}}-2\frac{s^{\prime }{\left(t\right)}^{2}}{s{\left(t\right)}^{3}}\right)\right)\text{.}$$

If $s\left(t\right)=\sigma ,\xi^{\prime }\left(t\right)=0$ and $\xi^{\prime \prime }\left(t\right)=2\gamma s^{\prime }{\left(t\right)}^{2}/\sigma >0$. Differentiation gives $\Delta^{\prime }\left(t\right)+t\Delta^{\prime \prime }\left(t\right)=\xi^{\prime }\left(t\right)+\eta^{\prime }\left(t\right)\Delta \left(t\right)+\eta \left(t\right)\Delta^{\prime }\left(t\right)$ and $2\Delta^{\prime \prime }\left(t\right)+t\Delta^{\prime \prime \prime }\left(t\right)=\xi^{\prime \prime }\left(t\right)+\eta^{\prime \prime }\left(t\right)\Delta \left(t\right)+2\eta^{\prime }\left(t\right)\Delta^{\prime }\left(t\right)+\eta \left(t\right)\Delta^{\prime \prime }\left(t\right)$, so that if $s\left(t\right)=\sigma ,\Delta \left(t\right)=\Delta^{\prime }\left(t\right)=\Delta^{\prime \prime }\left(t\right)=0$ and $\Delta^{\prime \prime \prime }\left(t\right)=\xi^{\prime \prime }\left(t\right)/t>0$, and the proof is complete. □

Theorem 8 *Let* ν≥−1 *. Then the elements of* $\left\{G_{\alpha ,\beta_{\nu }^{♭}\left(\alpha \right)}:\nu \leq \alpha \leq \alpha_{\nu }^{♭}\right\}$ *are mutually incomparable.*

Proof By Lemma 6, $\alpha \mapsto \alpha +\beta_{\nu }^{♭}\left(\alpha \right)$ is decreasing, whence the result by using Lemma 3. □

Theorem 9 *For* $\nu \geq -1/2,\alpha_{\nu }^{*}=\nu +1/2$ *and*

$$\beta_{\nu }^{*}\left(\nu +1/2\right)=\beta_{\nu }^{♭}\left(\nu +1/2\right)=\sqrt{\left(\nu +1/2\right)\left(\nu +3/2\right)}\text{.}$$

*For* $-1\leq \nu <-1/2,\alpha_{\nu }^{*}<\nu +1/2$*.*

Proof Let $\beta^{♭}=\beta_{\nu }^{♭}\left(\nu +1/2\right)=\sqrt{\left(\nu +1/2\right)\left(\nu +3/2\right)}$. For arbitrary β,

$$G_{\nu +1/2,\beta }=1-\frac{\nu +1/2}{t}+\frac{2{\left(\nu +1/2\right)}^{2}-\beta^{2}}{2t^{2}}+O\left(t^{-3}\right)\text{,}\;t\to \infty$$

by Eq. (4) and comparison with Eq. (1) shows that $G_{\nu +1/2,\beta }\geq R_{\nu }$ is only possible if

$$2{\left(\nu +1/2\right)}^{2}-\beta^{2}\geq \left(\nu +1/2\right)\left(\nu -1/2\right)\text{,}$$

or equivalently, if $\beta^{2}\leq 2{\left(\nu +1/2\right)}^{2}-\left(\nu +1/2\right)\left(\nu -1/2\right)=\left(\nu +1/2\right)\left(\nu +3/2\right)$. For ν<−1/2, the upper bound is negative, so that $G_{\nu +1/2,\beta }\geq R_{\nu }$ is impossible for all β≥0 and hence $\alpha_{\nu }^{*}<\nu +1/2$. For ν≥1/2, the condition is equivalent to $\beta \leq \beta^{♭}$. By Theorem 7, $\left(\nu +1/2,\beta^{♭}\right)\in U_{\nu }$ and by Theorem 1, α≤ν+1/2, so that $\alpha_{\nu }^{*}=\nu +1/2$ and $\beta_{\nu }^{*}\left(\nu +1/2\right)=\beta^{♭}$. □

Theorem 10 *Let* ν≥−1/2 *and* ν<α<ν+1/2 *. Then there exists a unique positive* $t_{\nu }^{*}\left(\alpha \right)$ *at which* $G_{\alpha ,\beta_{\nu }^{*}\left(\alpha \right)}$ *is tangent to* $R_{\nu }$ *. The map* $\alpha \mapsto t_{\nu }^{*}\left(\alpha \right)$ *is continuous and increasing on* (ν,ν+1/2)*, with* $\lim_{\alpha \to \nu +}t_{\nu }^{*}\left(\alpha \right)=0$ *and* $\lim_{\alpha \to \nu +1/2-}t_{\nu }^{*}\left(\alpha \right)=\infty$*.*

Proof Write $\beta^{*}=\beta_{\nu }^{*}\left(\alpha \right)$. By Theorem 6, we can find δ>0 such that $\beta^{*}\leq 2\left(\nu +1\right)-\alpha -\delta$. Using Lemma 3 and the fact that $\sqrt{\left(\nu +1/2\right)\left(\nu +3/2\right)}\leq \beta_{\nu }^{♭}\left(\alpha \right)\leq \beta^{*}$, we can find $0<t_{1}<t_{2}$ such that for all $\beta^{*}\leq \beta \leq \beta^{*}+\delta ,G_{\alpha ,\beta }\left(t\right)\geq G_{\nu ,\nu +2}\left(t\right)>R_{\nu }\left(t\right)$ for $0<t\leq t_{1}$ and $G_{\alpha ,\beta }\left(t\right)\geq G_{\nu +1/2,\sqrt{\left(\nu +1/2\right)\left(\nu +3/2\right)}}\left(t\right)>R_{\nu }\left(t\right)$ for $t\geq t_{2}$. If $G_{\alpha ,\beta^{*}}>R_{\nu }$, we have for all η>0 sufficiently small that $G_{\alpha ,\beta^{*}+\eta }\left(t\right)\geq R_{\nu }\left(t\right)$ for $t_{1}\leq t\leq t_{2}$. By the above, the same holds true for $0<t\leq t_{1}$ and $t\geq t_{2}$. Hence, $G_{\alpha ,\beta^{*}+\eta }\geq R_{\nu }$ for all η>0 sufficiently small, which contradicts the maximality of $\beta^{*}$. Thus, there must be at least one t>0 such that $G_{\alpha ,\beta^{*}}\left(t\right)=R_{\nu }\left(t\right)$, and clearly, the derivatives must agree at t as otherwise $G_{\alpha ,\beta^{*}}$ could not be an upper bound for $R_{\nu }$. Equivalently, $h_{\alpha ,\beta }$ must be tangent to $v_{\nu }$ at t. By Lemma 5, this is the case iff t solves $Q_{\alpha ,\beta^{*}}\left(\sqrt{t^{2}+\beta^{*2}}\right)=0$, from which we infer that $t=t_{\nu }^{*}\left(\alpha \right)$ is uniquely determined and continuous as a function of α. The limits for α→ν from the right and α→ν+1/2 from the left are obvious. To show that $t^{*}$ is increasing, it suffices to show that it is injective. Hence, let $\nu <\alpha_{1}<\alpha_{2}<\nu +1/2$ and suppose that $t_{\nu }^{*}\left(\alpha_{1}\right)=t_{\nu }^{*}\left(\alpha_{2}\right)=t^{*}$. Then with $\beta_{i}^{*}=\beta_{\nu }^{*}\left(\alpha_{i}\right)$, the $h_{\alpha_{i},\beta_{i}^{*}}$ must have the same value and derivative at $t^{*}$, so that

$$\frac{t^{*}}{\sqrt{t^{*2}+\beta_{1}^{*2}}}=\frac{t^{*}}{\sqrt{t^{*2}+\beta_{2}^{*2}}}\text{,}$$

and hence $\beta_{1}^{*}=\beta_{2}^{*}$, which is impossible as $\beta_{\nu }^{*}$ is decreasing by Theorem 5. □

Theorem 11 *Let* ν≥−1/2 *. Then* $\left\{G_{\alpha ,\beta_{\nu }^{*}\left(\alpha \right)}:\nu \leq \alpha \leq \nu +1/2\right\}$ *are the minimal elements of the family* $G_{U_{\nu }}$ *of upper Amos-type bounds for* $R_{\nu }$*, and*

$$R_{\nu }=\min\left\{G_{\alpha ,\overset{*}{\underset{\nu }{\beta }}\left(\alpha \right)}:\nu \leq \alpha \leq \nu +1/2\right\}\text{.}$$

Proof Let t>0. By Theorem 10, there exists a unique ν<α<ν+1/2 so that $t_{\nu }^{*}\left(\alpha \right)=t$ and hence $R_{\nu }\left(t\right)=G_{\alpha ,\beta_{\nu }^{*}\left(\alpha \right)}\left(t\right)$, proving the second assertion. Let $\nu \leq \alpha_{1}<\alpha_{2}\leq \nu +1/2$ and $\beta_{i}^{*}=\beta_{\nu }^{*}\left(\alpha_{i}\right)$. If $\alpha_{1}=\nu$, Theorem 6 shows that $2\left(\nu +1\right)=\alpha_{1}+\beta_{1}^{*}>\alpha_{2}+\beta_{2}^{*}$. If $\alpha_{1}>\nu$ and $\alpha_{1}+\beta_{1}^{*}\leq \alpha_{2}+\beta_{2}^{*}$, Lemma 3 implies that $R_{\nu }\leq G_{\alpha_{2},\beta_{2}^{*}}<G_{\alpha_{1},\beta_{1}^{*}}$, which is impossible as by Theorem 10, $G_{\alpha_{1},\beta_{1}^{*}}$ must be the only tangent to $R_{\nu }$ at $t_{\nu }^{*}\left(\alpha_{1}\right)$. Thus we always have $\alpha_{1}+\beta_{1}^{*}>\alpha_{2}+\beta_{2}^{*}$, and again by Lemma 3, there always exists $t=t\left(\alpha_{1},\alpha_{2}\right)$ such that $G_{\alpha_{1},\beta_{1}^{*}}\left(s\right)<G_{\alpha_{2},\beta_{2}^{*}}\left(s\right)$ for 0<s<t and $G_{\alpha_{1},\beta_{1}^{*}}\left(s\right)>G_{\alpha_{2},\beta_{2}^{*}}\left(s\right)$ for s>t. As $G_{\alpha ,\beta_{\nu }^{*}\left(\alpha \right)}>G_{\nu ,\nu +2}=G_{\nu ,\beta_{\nu }^{*}\left(\nu \right)}$ for α<ν and trivially $G_{\alpha ,\beta }\geq G_{\alpha ,\beta^{*}\left(\alpha \right)}$ provided that $\left(\alpha ,\beta \right)\in U_{\nu }$, the first assertion follows, and the proof is complete. □

Finally, let us consider the cases where ν=−k is a negative integer. As readily seen from the series expansion, $I_{-k}=I_{k}$, and hence $R_{-k}=I_{-k+1}/I_{-k}=I_{k-1}/I_{k}=1/R_{k-1}$.

Theorem 12 *If* k *is a positive integer,*

$$U_{-k}=\left\{\left(-\beta ,\beta \right):\beta \geq k\right\}$$

*and* $G_{-k,k}$ *is the minimum of the family* $G_{U_{\nu }}$ *of upper Amos-type bounds for* $R_{-k}$*.*

Proof As $R_{-k}>0$ and has a pole at t=0, the same must be true for upper bounds $G_{\alpha ,\beta }$ of $R_{-k}$, implying that necessarily α+β=0. As

$$G_{-\beta ,\beta }\left(t\right)=\frac{t}{\sqrt{t^{2}+\beta^{2}}-\beta }=\frac{t\left(\sqrt{t^{2}+\beta^{2}}+\beta \right)}{\left(t^{2}+\beta^{2}\right)-\beta^{2}}=\frac{\sqrt{t^{2}+\beta^{2}}+\beta }{t}=\frac{1}{G_{\beta ,\beta }\left(t\right)}\text{,}$$

we have $1/G_{\beta ,\beta }=G_{-\beta ,\beta }\geq R_{-k}=1/R_{k-1}$ iff $R_{k-1}\geq G_{\beta ,\beta }$, i.e.,  $\left(\beta ,\beta \right)\in L_{k-1}$. From the characterization of $L_{\nu }$ for ν≥−1 (Theorem 3), this is possible iff β≥k−1/2 and 2β≥2k, or equivalently, β≥k. □

Theorem 13 *If* k *is a positive integer,*

$$L_{-k}=\left\{\left(\alpha ,\beta \right):\alpha \geq -\left(k-1/2\right),\alpha +\beta \geq 0,\beta \geq 0\right\}$$

*and* $G_{-\left(k-1/2\right),k-1/2}$ *is the maximum of the family* $G_{L_{\nu }}$ *of lower Amos-type bounds for* $R_{-k}$*.*

Proof For lower bounds $G_{\alpha ,\beta }$ of $R_{-k}$, we must have α+β≥0 by the usual arguments, and Theorem 1 implies that necessarily α≥−k+1/2. On the other hand, we also know that $R_{k-1}\leq G_{k-1/2,k-1/2}$, or equivalently, $G_{-\left(k-1/2\right),k-1/2}\leq R_{-k}$, and the proof is complete. □

Note that for k=1, we already know by Theorem 3 that $G_{-1+1/2,-1+3/2}=G_{-1/2,1/2}$ is the greatest lower bound for $R_{-1}$, and Theorem 6 yields that $\beta_{-1}^{*}\left(-1\right)=1$, so that $G_{-1,1}$ is the least upper bound for $R_{-1}$ with α=−1.

## 5 Summary and conclusions

In this paper, we systematically investigate lower and upper Amos-type bounds for $R_{\nu }=I_{\nu +1}/I_{\nu }$ on the positive reals when $R_{\nu }$ is positive, or equivalently, when ν≥−1 or ν is a negative integer.

For ν≥−1, the set $L_{\nu }$ of all (α,β) giving lower bounds $G_{\alpha ,\beta }\leq R_{\nu }$ has a simple explicit description, and $G_{\nu +1/2,\nu +3/2}$ is the maximum of the family $G_{L_{\nu }}$ of lower Amos-type bounds for $R_{\nu }$ (Theorem 3).

For ν≥−1, the set $U_{\nu }$ of all (α,β) giving upper bounds $G_{\alpha ,\beta }\geq R_{\nu }$ is of the form $\left\{\left(\alpha ,\beta \right):\alpha \leq \alpha_{\nu }^{*},\max\left(0,-\alpha \right)\leq \beta \leq \beta_{\nu }^{*}\left(\alpha \right)\right\}$, where $\nu \leq \alpha_{\nu }^{*}\leq \nu +1/2$ and $\beta_{\nu }^{*}$ is continuous, decreasing and concave (Theorem 5), with $\beta_{\nu }^{*}\left(\nu \right)=\nu +2$ and $\alpha +\beta_{\nu }^{*}\left(\alpha \right)<2\left(\nu +1\right)$ for α>ν (Theorem 6). If ν≥−1/2, $\alpha_{\nu }^{*}=\nu +1/2$ and $\beta_{\nu }^{*}\left(\nu +1/2\right)=\sqrt{\left(\nu +1/2\right)\left(\nu +3/2\right)}$ by Theorem 9, and the upper bounds in the family $\left\{G_{\alpha ,\beta_{\nu }^{*}}\left(\alpha \right),\nu \leq \alpha \leq \nu +1/2\right\}$ are tangent to $R_{\nu }$ in exactly one point $t_{\nu }^{*}\left(\alpha \right)$ (Theorem 10, taking $t_{\nu }^{*}\left(\nu \right)=0$ and $t_{\nu }^{*}\left(\nu +1/2\right)=\infty$), and the minimal elements of the family $G_{U_{\nu }}$ of upper Amos-type bounds for $R_{\nu }$, with $R_{\nu }$ as their lower envelope (Theorem 11).

Thus, for ν≥−1, the pointwise maximum over all lower Amos-type bounds equals $G_{\nu +1/2,\nu +3/2}<R_{\nu }$, and hence is always smaller than $R_{\nu }$. On the other hand, for ν≥−1/2, the pointwise minimum over all upper Amos-type bounds equals  $R_{\nu }$.

For ν≥−1 and $\nu \leq \alpha <\alpha_{\nu }^{♭}=\min\left(\nu +1/2,2\nu +1\right)$, Theorems 7 and 8 establish a family $\left\{G_{\alpha ,\beta_{\nu }^{♭}}\left(\alpha \right),\nu \leq \alpha \leq \alpha_{\nu }^{♭}\right\}$ of explicitly computable, mutually incomparable upper bounds for $R_{\nu }$ with $\beta_{\nu }^{♭}\left(\nu \right)=\beta_{\nu }^{*}\left(\nu \right)=\nu +2$. For $\nu <\alpha <\alpha_{\nu }^{♭}$, these bounds are new. For $\nu \geq -1/2,\alpha_{\nu }^{♭}=\alpha_{\nu }^{*}=\nu +1/2$ and $\beta_{\nu }^{♭}\left(\nu +1/2\right)=\beta_{\nu }^{*}\left(\nu +1/2\right)$, and Theorem 7 extends the range of the bound $G_{\nu +1/2,\sqrt{\left(\nu +1/2\right)\left(\nu +3/2\right)}}\geq R_{\nu }$ given in Simpson and Spector [9] from ν≥0 to ν≥−1/2, and for −1/2<ν<0 dominates $G_{\nu +1/2,\nu +1/2}$ as the best previously available upper bound with α=ν+1/2 (and hence first order exact as t→∞).

Finally, for the cases where ν=−k is a negative integer, Theorems 12 and 13 give explicit characterizations of $U_{-k}$ and $L_{-k}$, and establish $G_{-k,k}$ and $G_{-\left(k-1/2\right),k-1/2}$ as the least upper and greatest lower Amos-type bounds for $R_{-k}$, respectively.

For −1≤ν<−1/2, the value of $\alpha_{\nu }^{*}$ is not known; the results in this paper imply that $\alpha_{\nu }^{♭}\leq \alpha_{\nu }^{*}<\nu +1/2$. It is also not known whether in this case $R_{\nu }$ can be obtained as the lower envelope of all upper Amos-type bounds. For ν=−1, this is certainly not the case (as $G_{-1,1}$ is the uniformly smallest upper bound). Hence the range −1<ν<−1/2 deserves further investigation.
